# Motor neuron degeneration correlates with respiratory dysfunction in SCA1

**DOI:** 10.1242/dmm.032623

**Published:** 2018-02-01

**Authors:** James P. Orengo, Meike E. van der Heijden, Shuang Hao, Jianrong Tang, Harry T. Orr, Huda Y. Zoghbi

**Affiliations:** 1 Department of Neurology, Baylor College of Medicine, Houston, TX 77030, USA; 2 Jan and Dan Duncan Neurological Research Institute at Texas Children's Hospital, Houston, TX 77030, USA; 3 Department of Neuroscience, Baylor College of Medicine, Houston, TX 77030, USA; 4 Department of Pediatrics, Baylor College of Medicine, Houston, TX 77030, USA; 5 Department of Laboratory Medicine and Pathology, University of Minnesota, Minneapolis, MN 55455, USA; 6 Department of Molecular and Human Genetics, Baylor College of Medicine, Houston, TX 77030, USA

**Keywords:** Motor neuron degeneration, SCA1, Spinocerebellar ataxia type 1, Bulbar dysfunction

## Abstract

Spinocerebellar ataxia type 1 (SCA1) is characterized by adult-onset cerebellar degeneration with attendant loss of motor coordination. Bulbar function is eventually impaired and patients typically die from an inability to clear the airway. We investigated whether motor neuron degeneration is at the root of bulbar dysfunction by studying SCA1 knock-in (*Atxn1^154Q/+^*) mice. Spinal cord and brainstem motor neurons were assessed in *Atxn1^154Q/+^* mice at 1, 3 and 6 months of age. Specifically, we assessed breathing physiology, diaphragm histology and electromyography, and motor neuron histology and immunohistochemistry. *Atxn1^154Q/+^* mice show progressive neuromuscular respiratory abnormalities, neurogenic changes in the diaphragm, and motor neuron degeneration in the spinal cord and brainstem. Motor neuron degeneration is accompanied by reactive astrocytosis and accumulation of Atxn1 aggregates in the motor neuron nuclei. This observation correlates with previous findings in SCA1 patient tissue. *Atxn1^154Q/+^* mice develop bulbar dysfunction because of motor neuron degeneration. These findings confirm the *Atxn1^154Q/+^* line as a SCA1 model with face and construct validity for this understudied disease feature. Furthermore, this model is suitable for studying the pathogenic mechanism driving motor neuron degeneration in SCA1 and possibly other degenerative motor neuron diseases. From a clinical standpoint, the data indicate that pulmonary function testing and employment of non-invasive ventilator support could be beneficial in SCA1 patients. The physiological tests used in this study might serve as valuable biomarkers for future therapeutic interventions and clinical trials.

This article has an associated First Person interview with the first author of the paper.

## Abstract (precis)

**Summary:** In this manuscript, we discovered motor neuron degeneration which correlates with respiratory failure in a knock-in mouse model of spinocerebellar ataxia type 1 (SCA1).

## INTRODUCTION

The spinocerebellar ataxias are so named because their most prominent symptoms are cerebellar: typically, a problem with balance or gait that appears in midlife. Spinocerebellar ataxia type 1 (SCA1) is paradigmatic of the spinocerebellar ataxias and of the particular family of polyglutamine diseases to which it belongs, including Huntington's disease and spinal and bulbar muscular atrophy (SBMA). These diseases result from expansion of a CAG repeat in the disease gene that encodes a polyglutamine (polyQ) tract in the resulting protein, which in the case of SCA1 is ATAXIN1 (Atxn1) (Chung et al., 1993). In individuals with SCA1 the disease progresses beyond balance difficulties and over a period of 10 to 30 years patients slowly develop spasticity, dysarthria, dysphagia, muscle wasting, ophthalmoplegia and a sensory neuropathy. Patients typically die from aspiration pneumonia, as it becomes impossible for them to keep their airway clear.

The majority of SCA1 symptoms arise from dysfunction of Purkinje neurons and brainstem neurons (Zoghbi and Orr, 1995). There are, however, also signs of motor neuron degeneration: early on some patients develop fasciculations, and as the disease progresses there can be hypo- or hyper-reflexia in addition to the spasticity and muscle wasting. Nerve conduction studies and electromyography (EMG) have revealed a primary motor neuronopathy with a smaller contribution of a dying-back axonopathy (van de Warrenburg et al., 2004; Linnemann et al., 2016). Dysphagia and dysarthria could arise from cerebellar dysfunction but could also, in principle, reflect motor neuron loss through lesions in cranial nerves, or the corticobulbar pathway. In fact, human SCA1 autopsy studies have demonstrated loss of motor neurons in the brainstem and spinal cord (Robitaille et al., 1995; Seidel et al., 2012; Kameya et al., 1995; Abe et al., 1996).

To determine whether motor neurons are involved in SCA1 bulbar dysfunction, here we employ the knock-in mouse model for SCA1, referred to as the *Atxn1^154Q/+^* line (Watase et al., 2002); this model has been used extensively to understand SCA1 pathogenesis. Previous focus has been on cerebellar dysfunction, as this is the prominent and presenting feature of the disease, and the particular vulnerability of the cerebellum to SCA1 has driven many studies (Jafar-Nejad et al., 2011; Fryer et al., 2011; Gennarino et al., 2015; Lasagna-Reeves et al., 2015a). The *Atxn1^154Q/+^* mice, like patients, show some signs that could indicate motor neuron disease, such as significant muscle wasting with progressive weight loss. To date, however, there has been only one report that specifically investigates motor neuron dysfunction in *Atxn1^154Q/+^* mice: Takechi and colleagues describe evidence of motor neuron degeneration in the spinal cord, with thin demyelinated axons, decreased nerve conduction velocities and reduced amplitudes of muscle action potentials (Takechi et al., 2013). Whether these findings correlate with the bulbar dysfunction that underlies early lethality, however, was not investigated.

Here, we examine the contribution of motor neuron degeneration to bulbar dysfunction in SCA1 and evaluate whether our model phenocopies this aspect of human disease (face validity) upon expression of the polyQ Atxn1 under its endogenous promotor (construct validity).

## RESULTS

### *Atxn1^154Q/+^* mice show progressive neuromuscular respiratory dysfunction

**Fig. 1.**
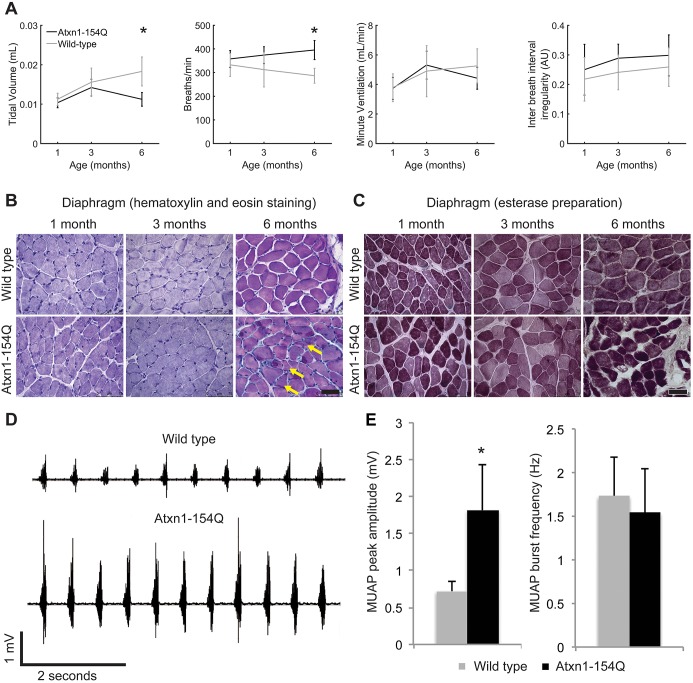
***Atxn1^154Q/+^*** **mice show progressive neuromuscular respiratory dysfunction with neurogenic changes in the diaphragm.** (A) Age-matched wild-type and *Atxn1^154Q/+^* mice (gray and black lines, respectively) were assessed by whole-body unrestrained plethysmography at 1, 3 and 6 months of age. Traces show, from left to right, average tidal volume, respiratory rate, minute ventilation and inter-breath interval irregularity, ±s.d. Statistical significance (*) between groups at 6 months was demonstrated using a two-way ANOVA (genotype×age), followed by a Tukey-Kramer post-hoc analysis (*P<0.05, *n*=15 mice per group). (B) Diaphragm muscles from *Atxn1^154Q/+^* and age-matched wild-type control mice at 1, 3 and 6 months of age were stained with Hematoxylin and Eosin. Diaphragms of 6 month old *Atxn1^154Q/+^* mice show a number of small angular fibers (arrows), consistent with a chronic neurogenic underlying process. (C) Diaphragms from *Atxn1^154Q/+^* mice and age-matched wild-type control mice at 1, 3 and 6 months of age were subjected to esterase enzyme histochemistry. *Atxn1^154Q/+^* mice at 6 months showed increased esterase activity (darker fibers), which indicates denervation. Scale bar: 50 μm. (D) Representative needle electromyography traces of diaphragms obtained from *Atxn1^154Q/+^* mice (lower trace) and age-matched wild-type control mice (upper trace) at 6 months of age. (E) Quantification of motor unit action potential (MUAP) amplitude and frequency illustrated as mean value in bar graphs±s.d. Statistical significance (*) between groups was demonstrated using a two-tailed Student's *t*-test (*P<0.05, *n*=4 mice per group).

To investigate whether SCA1 knock-in (*Atxn1^154Q/+^*) mice experience abnormal respiration, whole-body unrestrained plethysmography was employed. The respiratory parameters of *Atxn1^154Q/+^* mice and age- and sex-matched littermates were analyzed at 1, 3 and 6 months of age (15 mice per group). Compared with control mice, *Atxn1^154Q/+^* mice showed a progressive reduction in tidal volume, accompanied by a compensatory increase in respiratory rate, and no difference in inter-breath interval irregularity (Fig. 1A). By 6 months of age, *Atxn1^154Q/+^* mice had a significant reduction in tidal volume and increase in respiratory rate compared with both younger *Atxn1^154Q/+^* mice and age-matched littermate controls. *Atxn1^154Q/+^* mice compensate for the decrease in tidal volume by increasing their respiratory rate, thus preserving minute ventilation and sustaining life. However, at 6 months of age minute ventilation begins to trend downwards in the *Atxn1^154Q/+^* mice, indicating they are approaching respiratory failure. This pattern, together with unimpaired inter-breath interval regularity, highlight an intact regulatory breathing center in *Atxn1^154Q/+^* mice. This observation is not consistent with dysfunction driven by cerebellar pathology via signaling from vermal regions to the fastigial nucleus (Xu et al., 2004; Cao et al., 2012). *Atxn1^154Q/+^* mice thus develop respiratory dysfunction with a pattern consistent with neuromuscular pathology.

### Neurogenic changes drive dysfunction of the diaphragm in *Atxn1^154Q/+^* mice

Histology and electrophysiology were used to assess the integrity and function of the diaphragm muscle, which is responsible for inspiratory breathing movements. The diaphragm was collected from *Atxn1^154Q/+^* mice and control littermates at 1, 3 and 6 months of age (five mice per group). We processed the tissue using various stains to uncover any histopathological changes [Hematoxylin and Eosin (H&E) stain and esterase preparation shown in Fig. 1B and C, respectively].

As expected, wild-type sections at 1, 3 and 6 months of age were histologically normal. Tissues from *Atxn1^154Q/+^* mice were indistinguishable from wild type at early ages, but by 6 months of age histology revealed features consistent with denervation of the diaphragm. Recent denervation manifests as small angular-contoured muscle fibers (Engel, 2015), and we see an increased frequency of such fibers in the *Atxn1^154Q/+^* mice at 6 months of age (Fig. 1B, Fig. S1). Another characteristic of denervation present in *Atxn1^154Q/+^* mice at 6 months of age is the presence of fibers with increased esterase activity (Engel, 2015), illustrated as darker staining orange-brown fibers (Fig. 1C, Fig. S2). We observed no necrosis, regeneration or fibrosis, all characteristics of intrinsic muscle disease. There were rare findings of vacuolization of fibers in the *Atxn1^154Q/+^* mice at 6 months of age, but periodic acid-Schiff (PAS) and Oil Red O staining did not reveal any glycogen or lipids in these vacuoles (data not shown).

Diaphragm motor unit action potentials (MUAP) were recorded using needle EMG. This method enables the root cause of muscle dysfunction to be determined: that is, whether it is intrinsic to muscle disease (myopathic) or secondary to denervation (neuropathic) (Mills, 2005). Four mice per group were tested at 6 months of age. MUAP amplitude in *Atxn1^154Q/+^* mice was greater than twice that of age- and sex-matched littermate controls (Fig. 1D,E), confirming the respiratory dysfunction is due to an underlying motor nerve problem. There was no significant difference between groups in MUAP firing frequency, despite the increased respiratory rate in *Atxn1^154Q/+^* mice demonstrated on plethysmography testing. The reason for this discrepancy is secondary to the use of anesthesia, which diminishes the breathing center's ability to regulate respiratory rate, in combination with artificial ventilation that increases positive pressure to the lungs. Together, these findings confirm an underlying neurogenic process driving diaphragm dysfunction.

### *Atxn1^154Q/+^* mice show motor neuron degeneration in the spinal cord and hypoglossal nucleus

Given the bulbar dysfunction in SCA1 patients, and the neurogenic changes seen in the diaphragm of our mouse model, two upstream populations of motor neurons were examined: those that reside in the anterior horn of the cervical spinal cord (which directly innervate the diaphragm) (Nicaise et al., 2012) and those in the hypoglossal nucleus (nucleus of cranial nerve XII) in the brainstem, the disruption of which can produce dysarthria and dysphagia (Finsterer and Grisold, 2015). The hypoglossal nucleus has the advantage of being a pure motor nucleus without sensory neurons to confound interpretation of results.

**Fig. 2.**
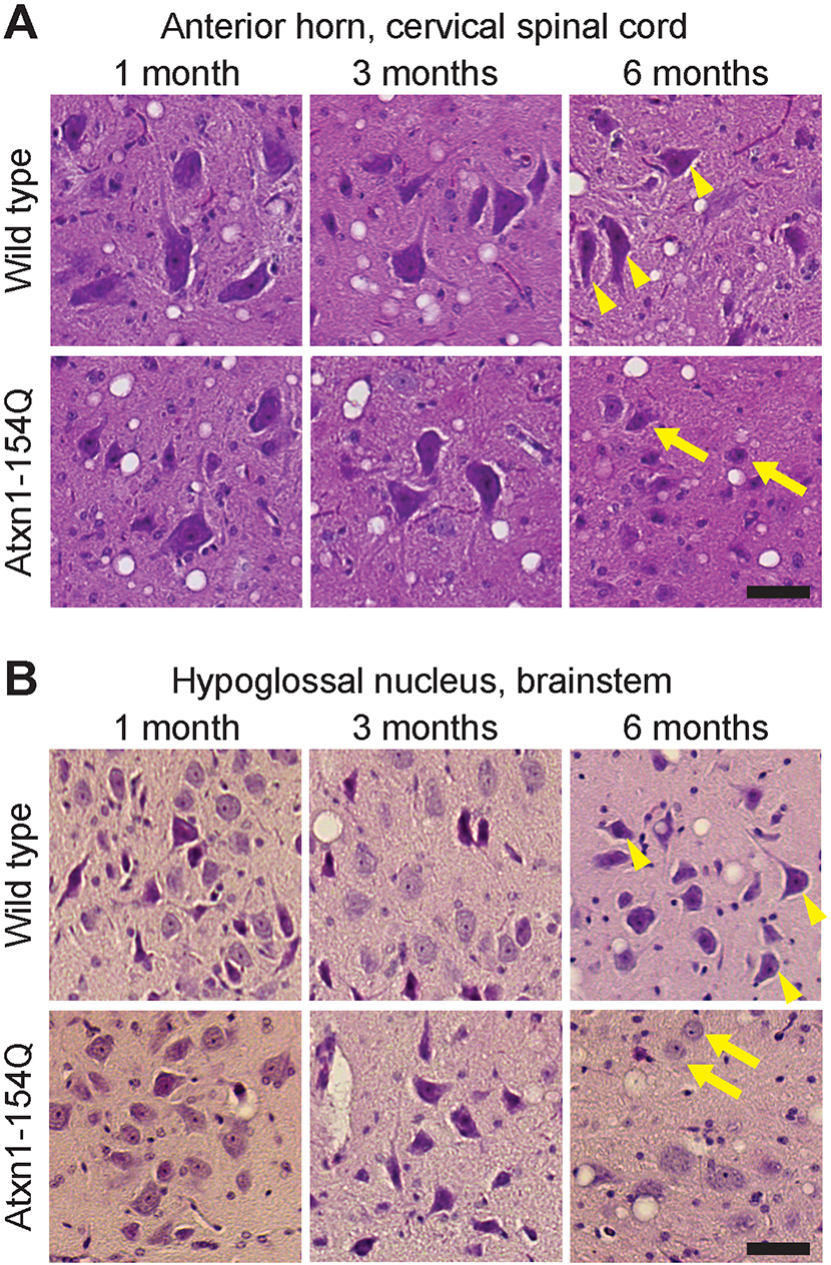
**Motor neuron degeneration in the spinal cord and hypoglossal nucleus.** Representative motor neurons from (A) the anterior horn of the cervical spinal cord and (B) the hypoglossal nucleus of the brainstem from *Atxn1^154Q/+^* mice and age-matched wild-type control mice at 1, 3 and 6 months of age, stained with Hematoxylin and Eosin. Arrowheads point to typical healthy motor neurons, which are pyramidal in shape. By contrast, motor neurons in the *Atxn1^154Q/+^* mice (arrows) become rounded and small by 6 months of age. Scale bar: 50 μm.

Motor neuron integrity was assessed using H&E staining on paraffin-embedded tissue from SCA1 mice and wild-type controls at 1, 3 and 6 months of age. No abnormalities were found in motor neurons in the cervical spinal cord or hypoglossal nucleus in *Atxn1^154Q/+^* mice at the younger ages. We did, however, find morphologically abnormal neurons by 6 months of age in the *Atxn1^154Q/+^* mice that distinguished them from both younger *Atxn1^154Q/+^* mice and wild-type mice of any age. These motor neurons had lost their pyramidal shape, becoming more rounded, and eventually shrank in size (Fig. 2, Fig. S3) (Ahmad et al., 2012; Saeri et al., 2015). The resulting dysfunction of these neurons probably explains the progressive respiratory dysfunction observed in these mice. More importantly, this degeneration of motor neurons in the spinal cord and hypoglossal nucleus recapitulates that reported in human patients with SCA1 (Robitaille et al., 1995; Seidel et al., 2012; Kameya et al., 1995; Abe et al., 1996), reinforcing the notion that motor neuron degeneration has a role in SCA1 dyspnea, dysphagia and dysarthria.

### *Atxn1^154Q/+^* mice demonstrate reactive astrocytosis in the spinal cord and hypoglossal nucleus

**Fig. 3.**
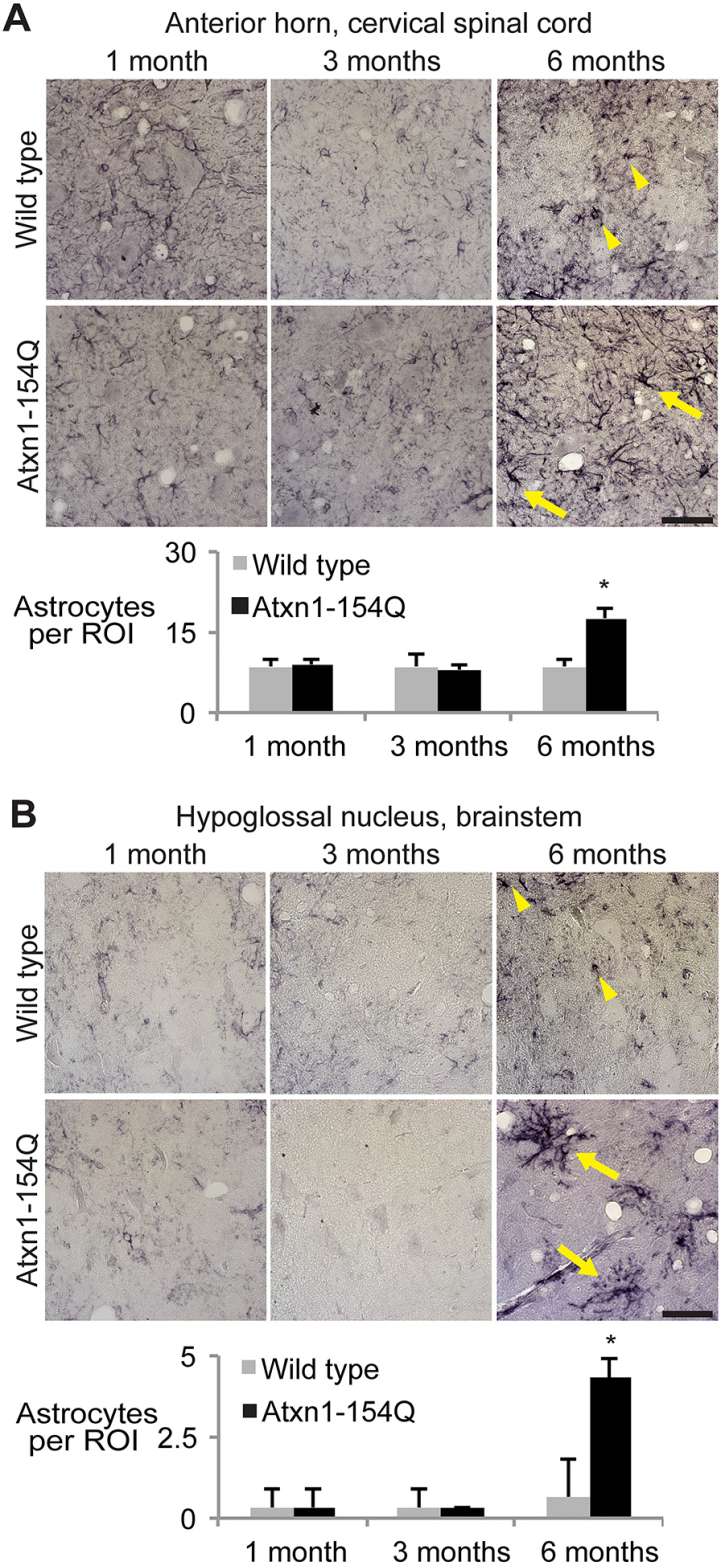
**Reactive astrocytosis in motor neuron areas of aged *Atxn1^154Q/+^* mice.** Diaminobenzidine (DAB) immunohistochemistry was performed with an antibody against glial fibrillary astrocyte protein in paraffin-embedded tissue from *Atxn1^154Q/+^* mice and age-matched wild-type control mice at 1, 3 and 6 months of age. Arrowheads highlight astrocytes in areas of healthy motor neurons. By 6 months of age, the *Atxn1^154Q/+^* astrocytes show a significant increase in density as well as notable reactive hypertrophy (arrows) in (A) the anterior horn of the cervical spinal cord and (B) the hypoglossal nucleus of the brainstem. Astrocyte density was determined by counting the number of astrocytes per region of interest (ROI, defined as an area of 46,225 μm^2^). Bars illustrate the mean number of astrocytes per ROI±standard deviation. Statistical significance between all groups and *Atxn1^154Q/+^* mice at 6 months was demonstrated using a two-way ANOVA (genotype×age), followed by a Tukey-Kramer post-hoc analysis (*P<0.05, *n*=3 mice per group). Scale bar: 50 μm.

To determine whether the degenerating neurons elicit a response from astrocytes, we surveyed the anterior horn of the cervical spinal cord and the hypoglossal nucleus for reactive astrocytosis using an antibody to glial fibrillary astrocyte protein (GFAP) (Bruijn and Cleveland, 1996). No differences were observed between wild-type mice and *Atxn1^154Q/+^* mice at younger ages in either region, but there was noticeable astrocyte proliferation and hypertrophy by 6 months of age in the *Atxn1^154Q/+^* mice (Fig. 3). The progressive motor neuron dysfunction that occurs in these mice between 3 and 6 months of age thus elicits reactive astrocytosis.

### *Atxn1^154Q/+^* protein forms intranuclear aggregates in motor neurons of the spinal cord and hypoglossal nucleus

**Fig. 4.**
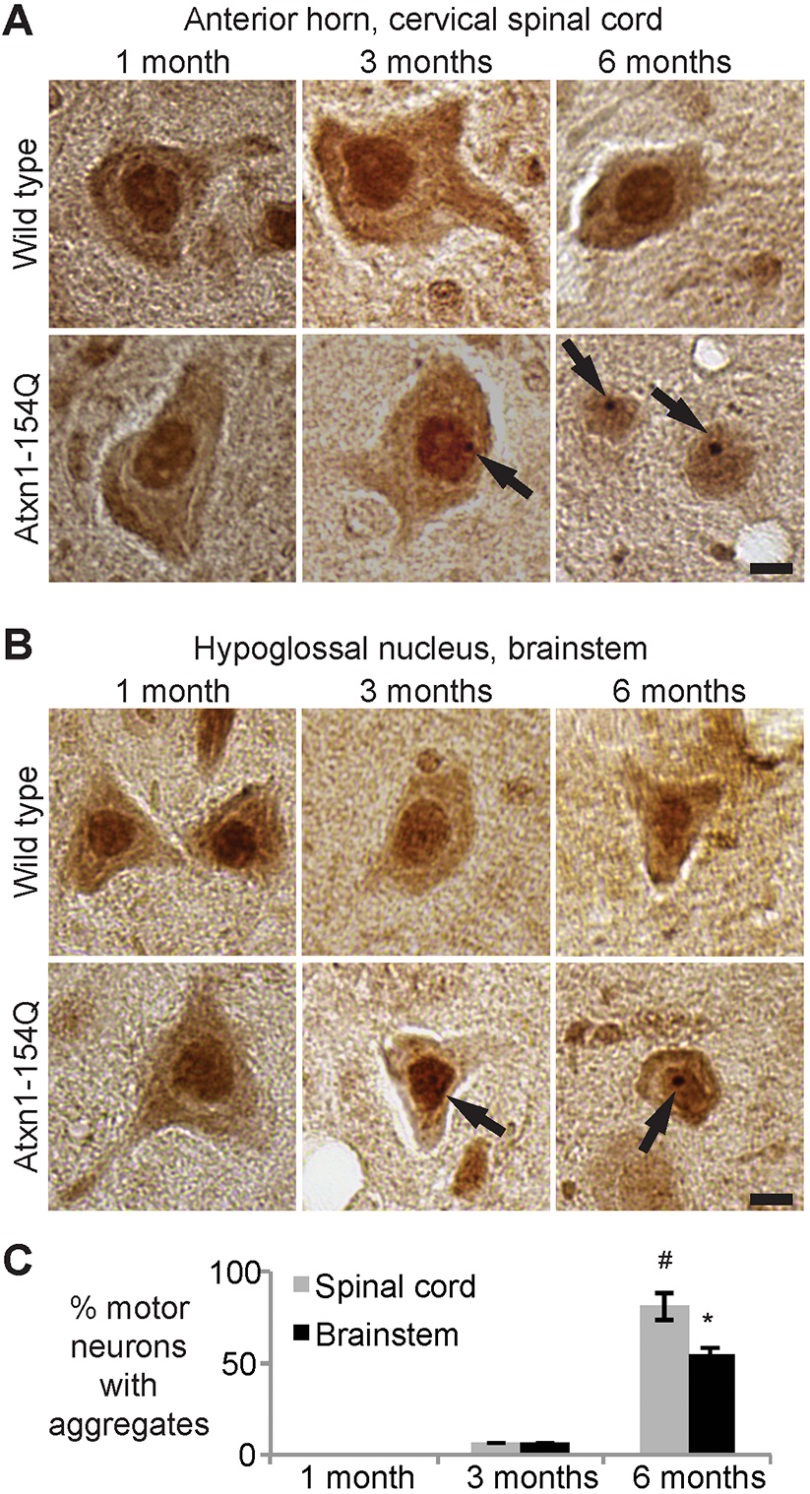
**Age-dependent formation of Atxn1 nuclear inclusions in motor neurons of *Atxn1^154Q/+^* mice.** Representative motor neurons from (A) the anterior horn of the cervical spinal cord and (B) the hypoglossal nucleus of the brainstem. DAB immunohistochemistry was performed with an antibody against Atxn1 in paraffin-embedded tissue from *Atxn1^154Q/+^* mice and age-matched wild-type control mice at 1, 3 and 6 months of age. Motor neurons in the *Atxn1^154Q/+^* mice develop sparse, small nuclear aggregates at 3 months of age, which increase in size and frequency by 6 months of age (arrows). Scale bar: 10 μm. (C) Quantification of the number of motor neurons with detectable nuclear aggregates of Atxn1 protein in *Atxn1^154Q/2^* mice at 40× magnification. A total of 100-200 motor neurons were counted per animal and averages calculated for three mice per group. Bars illustrate mean±s.d. for each group. Statistical significance between 3 and 6 months for spinal cord (#) and brainstem (*) was demonstrated using a two-tailed Student's *t*-test (*P*<0.05).

Polyglutamine expansions in the Atxn1 protein promote its accumulation into intranuclear inclusions in various groups of neurons. The timing of aggregate formation varies significantly on the basis of cell type; for example, in *Atxn1^154Q/+^* mice aggregates form robustly in cortical neurons as early as 6 weeks, although these aggregates do not begin to appear until 20 weeks of age in Purkinje neurons and do not become prevalent until 40 weeks (Watase et al., 2002). Therefore, we set out to determine if, and when, Atxn1 nuclear aggregates form in motor neurons of the cervical spinal cord and hypoglossal nucleus. In *Atxn1^154Q/+^* mice, intranuclear inclusions began to appear as early as 3 months of age in motor neurons: 5.8% of motor neurons in the spinal cord and 5.7% in the hypoglossal nucleus had visible aggregates (Fig. 4). By 6 months of age, 80.8% of motor neurons in the spinal cord and 55% of motor neurons in the hypoglossal nucleus had developed visible aggregates.

## DISCUSSION

Here, we show that *Atxn1^154Q/+^* mice are a physiologically accurate model for studying motor neuron degeneration and the more prominent cerebellar degeneration of SCA1, having both face and construct validity. Unlike many mouse models of neurodegenerative diseases, in which the mutant human proteins need to be markedly overexpressed (often using heterologous promoters) in order to produce a phenotype within the relatively short life-span of the mouse, the *Atxn1^154Q/+^* mouse bears 154 CAG repeats in the endogenous mouse locus. Granted, this repeat expansion is longer than that seen in humans, and thus causes a more severe, rapidly progressing disease, but the spatio-temporal expression of the expanded Atxn1 protein is physiologically comparable to the human disease, as is the phenotype.

This mouse model has been used successfully in a variety of studies to understand SCA1 pathogenesis. Our laboratory has evaluated transcriptional alterations that occur early in the disease and discovered that at least one reason the Purkinje neurons seem to be particularly vulnerable in SCA1 is because they express a protein partner of Atxn1, Capicua, at particularly high levels. The polyglutamine expansion intensifies this interaction, causing a strong gain-of-function within these cells. Reducing Capicua levels largely rescues Purkinje neuron degeneration (Lam et al., 2006; Fryer et al., 2011); however, the life span of the mice remains short, suggesting that there is another cell population and molecular pathway important for promoting premature death. In patients, bulbar dysfunction is directly involved in SCA1 lethality, as failure to clear the airway leads to aspiration pneumonia. The current study shows that bulbar dysfunction and concomitant respiratory complications are rooted in the degeneration of motor neurons.

Patients with other polyglutamine diseases, such as SCA2 and SCA3, have also been reported to display motor neuron degeneration. Moreover, diseases caused by non-coding expansions, such as SCA36, and hexanucleotide expansions, such as C9ORF72 amyotrophic lateral sclerosis (ALS)/fronto-temporal dementia, also tend to involve motor neuron degeneration (Nanetti et al., 2009; Rosenberg, 1992; Watanabe et al., 1996; Ikeda et al., 2012). In addition, SCA2 patients with intermediate length CAG repeats, which are not quite sufficient to drive cerebellar degeneration, are at increased risk for developing ALS (Sproviero et al., 2017). Unraveling the molecular pathways leading to motor neuron degeneration in the SCA1 mouse model might prove useful in understanding these other disorders.

Our findings also have ramifications for the clinical care of SCA1 patients. We have established a series of assays (plethysmography and needle EMG) that are minimally invasive and independent of cerebellar function, which could be used as biomarkers in our mouse model to follow outcomes of novel therapeutic trials aimed at reducing motor neuron degeneration. We recommend following these same outcome parameters in clinical trials in SCA1 patients, in addition to using cerebellar function scores. Finally, clinicians should routinely follow pulmonary function tests in SCA1 patients, and possibly in patients with other spinocerebellar ataxias with documented motor neuron involvement. This population could benefit from early intervention with non-invasive ventilator support, as does the ALS population (Hobson and McDermott, 2016).

## MATERIALS AND METHODS

### Whole body plethysmography

Breathing behavior was tested as described previously (Huang et al., 2012, 2016; Ward et al., 2011; Yeh et al., 2017). Breathing physiology was assessed using whole-body plethysmography (Buxco). *Atxn1^154Q/+^* mice and littermate controls were tested at 1, 3 and 6 months of age (*n*=15 mice per group, approximating a male:female ratio of 2:3). Subgroup analysis demonstrated no significant difference between males and females in any group (data not shown). These time points were chosen because *Atxn1^154Q/+^* mice are presymptomatic at 1 month of age, show reliable behavioral signs of cerebellar dysfunction at 3 months, and are nearing humane end-point at 6 months of age (Watase et al., 2002). Mice were habituated to the chambers for at least 1 h before recording respiration for 30 min. Fresh room air was pumped through the chambers at a continuous rate of 0.5 l/min. Breath waveforms were identified using Phonemah 3 software (DSI), and derived parameters including breathing rate, tidal volume and minute ventilation were analyzed and processed using customized MATLAB (MathWorks) code. To reduce artifacts from moving or sniffing, we excluded breaths with an inspiratory time under 0.025 s, expiratory time over 10 s or calculated expiratory tidal volume more than twice the inspiratory tidal volume. Furthermore, a sliding window of 200 breaths was used to filter out intervals during which more than 10% of breaths were taken at a rate faster than 600 breaths/min. Inter-breath interval irregularity (IBII) was defined as: IBII=abs[breath length(*n*+1)−breath length(*n*)]/breath length(*n*), where abs is the absolute value and *n* is the chronological number of a recorded breath. Only mice from which it was possible to reliably record over 100 breaths were included in the analysis. Statistical significance was tested using a two-way ANOVA (genotype×age), followed by Tukey-Kramer post-hoc analysis. Significance was accepted at *P*-values <0.05. The protocol for this study has received approval by the Institutional Animal Care and Use Committee, and all studies were conducted in accordance with the United States Public Health Service's Policy on Humane Care and Use of Laboratory Animals.

### Electromyography

Mice were anesthetized with 1.5% isofluorane gas in oxygen and head-fixed on a stereotaxic frame. A 26 gauge EMG needle (AMBU, Malaysia) was inserted through the skin, underneath the last rib into the thoracic diaphragm. Bioelectrical signals were amplified at a gain of 100, filtered between 10 Hz and 5 kHz (Model 1700 Differential AC Amplifier, A-M Systems) and digitized at 10 kHz (1440A digitizer and pClamp 10, Molecular Devices). With the help of an audio monitor (Model 3300, A-M Systems), recordings were initiated when the point of maximal MUAP was identified and each recording period lasted at least 60 s. The maximal amplitudes of MUAP bursts and the burst frequencies were quantified from each recording session and then averaged to obtain the single values of the MUAP amplitude and frequency for each animal, respectively.

### Histology

Mouse tissue was collected and fixed for 24 h in 10% formalin. Standard techniques were used to embed tissue in paraffin and to take 10 μm sections in serial section every 100 μm. Slides were then stained with H&E. Special stains including esterase, succinate dehydrogenase, trichrome, Oil Red O and periodic acid-Schiff were performed on unfixed tissue snap-frozen with liquid nitrogen (Thompson, 1966; Sheehan and Hrapchak, 1987). Brightfield images were acquired using a Carl Zeiss Axio Imager M2 microscope, equipped with an Axio Cam MRc5 color camera (Carl Zeiss, Germany). All histology experiments were performed in triplicate; figures show representative results. For quantification, small angular contoured fibers or darkly staining esterase fibers were counted from a total of over 200 myofibers sampled per mouse. Motor neuron area was determined using ImageJ software, adjusting the image setting to a threshold of 70% and then sampling ten motor neurons per mouse.

### Immunohistochemistry

Immunohistochemistry was performed on paraffin-embedded sections. In brief, 10 μm sections were deparaffinized and rehydrated. Slides were treated with antigen retrieval by boiling for 9 min in 10 mM sodium citrate, 0.05% Tween 20, pH 6.0. Primary antibodies were detected with biotinylated goat anti-mouse IgG (1:1000) or biotinylated goat anti-rabbit IgG (1:1000) (Jackson ImmunoResearch Laboratories) and visualized using an ABC reagent kit (Vector Laboratories) according to the manufacturer's recommendations. The Atxn1 11NQ antibody, generated in our laboratory (Lasagna-Reeves et al., 2015b), was incubated at a concentration of 1:1000 for approximately 12 h at 4°C. The GFAP antibody (mouse monoclonal Sigma-Aldrich G3893) was used at a concentration of 1:500 and incubated for approximately 12 h at 4°C. All immunohistochemistry experiments were performed in triplicate and figures show representative results.

## Supplementary Material

Supplementary information

## Supplementary Material

First Person interview
