# Supplementary material for: Motor neuron degeneration correlates with respiratory dysfunction in SCA1
Source: Dis Model Mech. 2018 Feb 1;11(2):dmm032623. doi: 10.1242/dmm.032623 (PMC5894948; doi:10.1242/dmm.032623)
Supplement: Supplementary information [file dmm-11-032623-s1.pdf]

Supplemental Figure 1

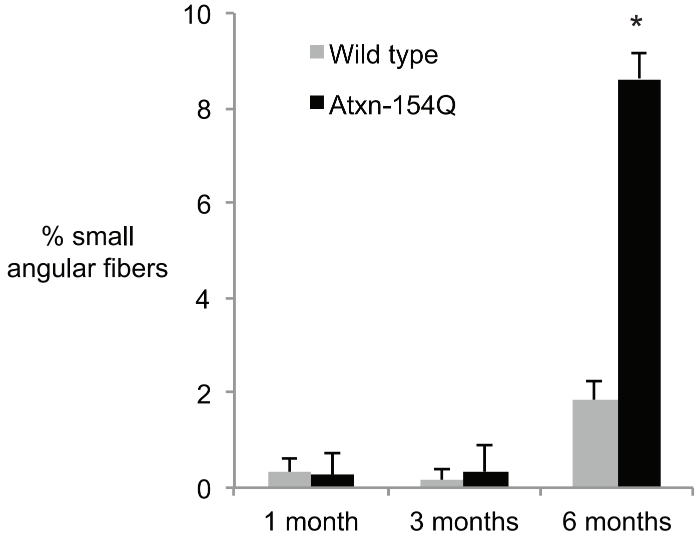

Supplementary Figure 1: Quantification of small angular fibers in diaphragm. As described in Figure 1B, diaphragms from Atxn1^{154Q} and age-matched wild-type control mice at 1, 3, and 6 months of age were stained with hematoxylin and eosin. Bar graph quantitates the mean percentage of small angular fibers per > 200 myofibers counted, +/- standard deviation, with N = 3 mice per genotype and time-point. Statistical significance between all groups and Atxn1^{154Q} mice at 6 months was demonstrated using a two-way ANOVA (genotype × age), followed by a Tukey-Kramer post-hoc analysis (* $p < 0.01$).

Supplemental Figure 2

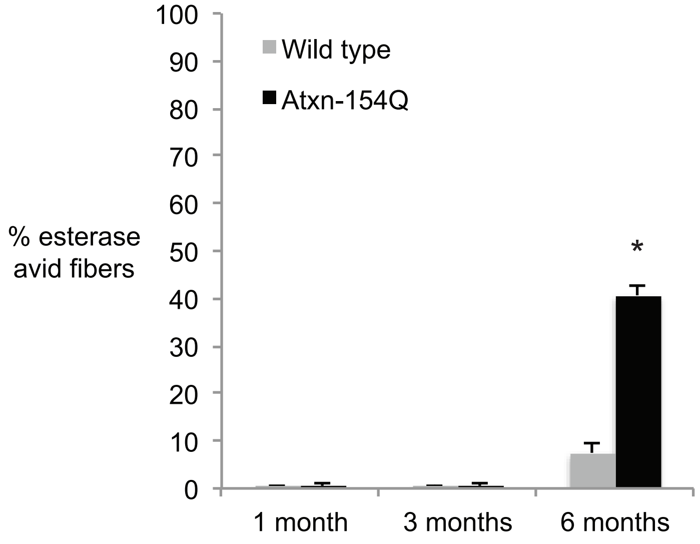

Supplementary Figure 2: Quantification of intensity of esterase staining in diaphragm fibers. As described in Figure 1C, diaphragms from Atxn1^{154Q} mice and age-matched wild-type control mice at 1, 3, and 6 months of age were subjected to esterase enzyme histochemistry, darker fibers indicate increased esterase activity and are denoted “esterase avid”. Bar graph quantitates the mean percentage of darkly staining fibers compared to baseline staining fibers, +/- standard deviation, with N = 3 mice per genotype and time-point. Statistical significance between all groups and Atxn1^{154Q} mice at 6 months was demonstrated using a two-way ANOVA (genotype × age), followed by a Tukey-Kramer post-hoc analysis (* $p < 0.01$).

Supplemental Figure 3

A

Anterior horn, cervical spinal cord

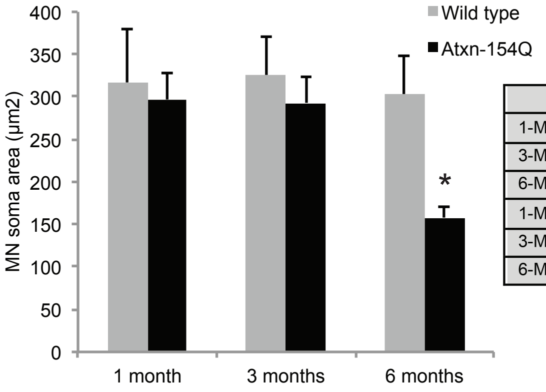

	1-MO_WT	3-MO_WT	6-MO_WT	1-MO_KI	3-MO_KI	6-MO_KI
1-MO_WT		0.9999	0.9973	0.9889	0.9754	0.0045
3-MO_WT	0.9999		0.9831	0.9576	0.9274	0.0031
6-MO_WT	0.9973	0.9831		1	0.9997	0.0092
1-MO_KI	0.9889	0.9576	1		1	0.0121
3-MO_KI	0.9754	0.9274	0.9997	1		0.0148
6-MO_KI	0.0045	0.0031	0.0092	0.0121	0.0148	

B

Hypoglossal nucleus, brainstem

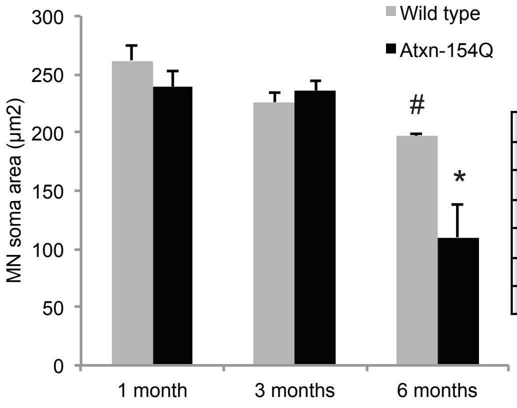

	1-MO_WT	3-MO_WT	6-MO_WT	1-MO_KI	3-MO_KI	6-MO_KI
1-MO_WT		0.1015	0.0017	0.4719	0.3235	0
3-MO_WT	0.1015		0.2107	0.881	0.967	0
6-MO_WT	0.0017	0.2107		0.037	0.0616	0.0001
1-MO_KI	0.4719	0.881	0.037		0.9996	0
3-MO_KI	0.3235	0.967	0.0616	0.9996		0
6-MO_KI	0	0	0.0001	0	0	

Supplementary Figure 3: Quantification of motor neuron diameter. As described in Figure 2, cervical spinal cord (A) and brainstem (B) from Atxn1^{154Q} mice and age-matched wild-type control mice at 1, 3, and 6 months of age were stained with hematoxylin and eosin. Motor neurons area (µm^2) was measured using image J software. Bar graphs quantitate the mean motor neuron (MN) area for each tissue, with N = 3 mice per genotype and time-point. Statistical significance between all groups and Atxn1^{154Q} mice at 6 months was demonstrated using a two-way ANOVA (genotype × age), followed by a Tukey-Kramer post-hoc analysis (* $p< 0.01$). Statistical significance between both groups at 1 month and wild-type mice at 6 months was determined using a two-way ANOVA (genotype × age), followed by a Tukey-Kramer post-hoc analysis (# $p< 0.01$). Tables represent calculated p values, for wild-type (WT) and Atxn1^{154Q} (KI) mice at all three time points.
