# Supplementary material for: Motor neuron degeneration correlates with respiratory dysfunction in SCA1
Source: Dis Model Mech. 2018 Feb 1;11(2):dmm032623. doi: 10.1242/dmm.032623 (PMC5894948; doi:10.1242/dmm.032623)
Supplement: First Person interview [file dmm-11-032623-s2.pdf]

FIRST PERSON

First person – James Orengo

First Person is a series of interviews with the first authors of a selection of papers published in Disease Models & Mechanisms, helping early-career researchers promote themselves alongside their papers. James Orengo is first author on 'Motor neuron degeneration correlates with respiratory dysfunction in SCA1', published in DMM. James is Assistant Professor of Neurology in the lab of Huda Zoghbi at Baylor College of Medicine, Houston, USA, investigating the biology of motor neuron degeneration.

How would you explain the main findings of your paper to non-scientific family and friends?
We are using a mouse model to better understand the cause of premature death in patients with a neurodegenerative disease that runs in families.

What are the potential implications of these results for your field of research?
Our paper shines light into the role of other brain areas outside of the cerebellum that are affected in SCA1. In particular, we found that a mouse model of SCA1 likely passes away from declining respiratory functions, which correlates with motor neuron degeneration.

"More opportunities to showcase early investigators' work early in solid journals is valuable as it can set the basis for further studies and provides some momentum to expand the project and obtain funding."

What are the main advantages and drawbacks of the model system you have used as it relates to the disease you are investigating?
We use a knock-in mouse model to study SCA1. The major advantage of this model is that expression of the pathogenic SCA1 protein occurs at physiological levels in a natural temporal and spatial pattern. When studying neurodegenerative diseases, which take decades of expression of pathogenic proteins to have an effect on cell death, using a short lifespan organism (mouse) as a model can be a drawback in demonstrating the full extent of disease.

What has surprised you the most while conducting your research?
The resilience of mice to overcome and/or adapt to a toxic perturbation in humans is very intriguing me.

James Orengo's contact details: Department of Neurology, Baylor College of Medicine, Houston, USA.
E-mail: James.Orengo@bcm.edu

This is an Open Access article distributed under the terms of the Creative Commons Attribution License (http://creativecommons.org/licenses/by/3.0), which permits unrestricted use, distribution and reproduction in any medium provided that the original work is properly attributed.

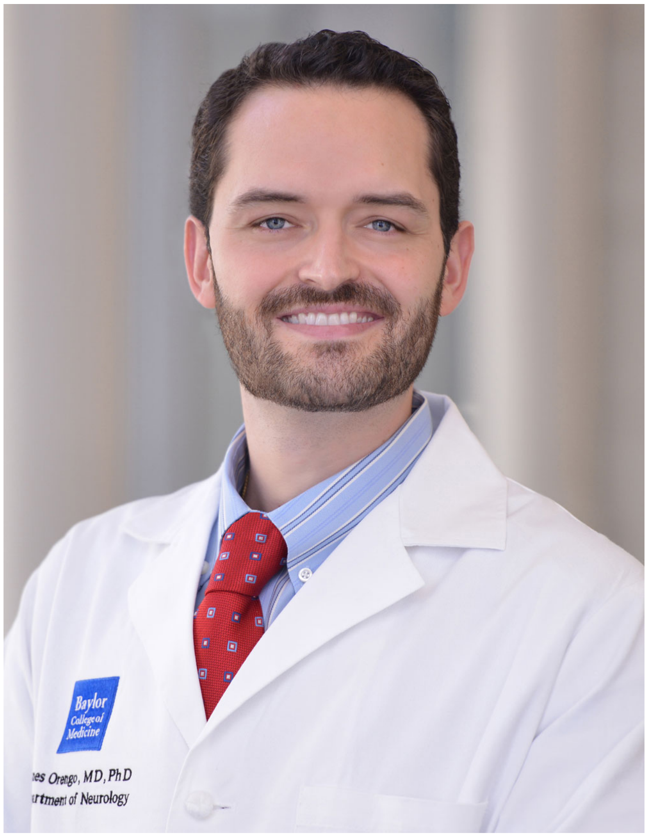

James Orengo

Describe what you think is the most significant challenge impacting your research at this time and how will this be addressed over the next 10 years?
The greatest hurdle to my research program at present is being able to study molecular events *in vivo* and in isolation within motor neurons. Exciting improvements in technology such as the development of high-resolution light microscopy from investigators such as Ed Boyden at MIT will greatly improve access.

What changes do you think could improve the professional lives of early-career scientists?
More opportunities to showcase early investigators' work early in solid journals is valuable as it can set the basis for further studies and provides some momentum to expand the project and obtain funding.

What's next for you?
I am currently developing a novel mouse model for spinocerebellar ataxia type 1 that will allow me to study cell-specific effects from expression of the mutant disease protein.

Reference

Orengo, J. P., van der Heijden, M. E., Hao, S., Tang, J., Orr, H. T. and Zoghbi, H. Y. (2018). Motor neuron degeneration correlates with respiratory dysfunction in SCA1. *Dis. Model. Mech.* **11**, doi:10.1242/dmm.032623.
